# Retraction: Molecular docking and dynamics simulation study of bioactive compounds from *Ficus carica* L. with important anticancer drug targets

**DOI:** 10.1371/journal.pone.0343359

**Published:** 2026-02-23

**Authors:** The PLOS One Editors

The *PLOS One* Editors retract this article [1] because it was identified as one of a series of submissions for which we have concerns about compromised peer review and compliance with PLOS policy on Manipulation of the Publication Process.

MAA did not agree with the retraction. ABG, JL, MAF, and KMAA either did not respond directly or could not be reached.
